# Modeling of the geographical distribution of two forest‐forming fucoids in the Strait–Alboran demarcation (southern Iberian Peninsula): Implications for conservation and restoration

**DOI:** 10.1111/jpy.70209

**Published:** 2026-07-31

**Authors:** Pablo Pérez‐Castro, Elena Bañares‐España, Antonio Flores‐Moya, Ignacio J. Melero‐Jiménez

**Affiliations:** ^1^ Departamento de Botánica y Fisiología Vegetal Universidad de Málaga, Andalucía Tech Málaga Spain

**Keywords:** Alboran Sea, climate change, fucoid algae, habitat suitability, marine forests, Mediterranean Sea, species distribution models, stratification

## Abstract

Marine forests formed by canopy‐forming brown algae of the former *Cystoseira* complex are key habitat‐forming components of Mediterranean coastal ecosystems, supporting biodiversity and multiple ecosystem functions. However, many populations have declined in recent decades due to increasing anthropogenic pressures and climate change, raising concerns about the persistence of these foundational species. Identifying the environmental drivers that shape their distribution is therefore essential for predicting future changes and guiding conservation actions. Here, we applied species distribution models to evaluate the environmental determinants, present habitat suitability, and potential future distribution of two emblematic fucoid species, *Gongolaria usneoides* and *Ericaria selaginoides*, in the Strait of Gibraltar–Alboran Sea region, a major Mediterranean biodiversity hotspot and climatic transition zone. Present‐day models were projected to the end of the century under two contrasting climate scenarios (SSP1‐1.9 and SSP5‐8.5). Mixed layer depth was the dominant predictor structuring species distributions, highlighting the role of water column stratification and hydrographic dynamics in regulating benthic habitats. The two species exhibited contrasting responses: *Gongolaria usneoides* occupied a narrow environmental niche and showed spatially heterogeneous future responses, including localized suitability increases around the Strait of Gibraltar and western Alboran sectors together with suitability declines across portions of the eastern coastline under both climate scenarios. In contrast, *Ericaria selaginoides* showed broader environmental tolerance and generally positive, although spatially heterogeneous, suitability responses across large portions of the region. These results suggest that future environmental change may restructure Mediterranean marine forests through species‐specific responses rather than through uniform decline.

**Abbreviations**
AUC: area under the curve
ESAL: Strait–Alboran demarcation area
GBIF: Global Biodiversity Information Facility
M: species‐specific accessible areas
MaxENT: Maximum Entropy Modeling
MLD: mixed layer depth
ROC: receiver operating characteristic
SDM: Species Distribution Model
SSP: Shared Socioeconomic Pathway

## INTRODUCTION

Marine forests of the brown seaweeds of the genus *Cystoseira* s.l. (recently divided into *Cystoseira*, *Ericaria*, and *Gongolaria*; Molinari Novoa & Guiry, 2020) are foundational components of the infralittoral in the Mediterranean and adjacent Atlantic waters (Báez et al., 2004; Báez, Olivero, et al., 2005; Báez, Real, et al., 2005; Bruno De Sousa et al., 2019; Hereu et al., 2008; Orellana et al., 2019; Rendina et al., 2023; Sales & Ballesteros, 2010). These forest‐forming fucoids provide complex habitats that support high levels of biodiversity and contribute significantly to ecosystem functioning (Ballesteros, 1990; Falace et al., 2018; Sales & Ballesteros, 2010). However, their persistence is increasingly jeopardized by the combined pressures of anthropogenic disturbance and climate change (Sales et al., 2011; Wernberg et al., 2016), which have led to widespread declines throughout the Mediterranean (Blanfuné et al., 2025; Clausing et al., 2024; Sales & Ballesteros, 2010; Thibaut et al., 2005). For these reasons, the European Union (EU) recognized the vulnerability of its forests by including them as habitats of community interest in the Habitats Directive (92/43/EEC, 1992), underscoring the need for active conservation measures (Falace et al., 2018). In the Spanish legislation, all species of the genus *Cystoseira* s.l., except *C. compressa,* were recorded in the List of Wild Species under Special Protection and the Spanish Catalogue of Threatened Species (RD 139/2011).

The decline of the marine forests of *Cystoseira* s.l. entails far‐reaching ecological consequences, including reduced biodiversity, compromised coastal protection, and loss of ecosystem services such as trophic support, nutrient cycling, and carbon sequestration (Álvarez‐Canali et al., 2019; Falace et al., 2018; Iveša et al., 2016). Drivers such as habitat fragmentation, population isolation, rising sea temperatures and alien species arrival have been linked to population declines and range contraction, reduced genetic connectivity, and altered reproductive phenology in *Cystoseira* s.l. populations, hampering natural recolonization and range expansion (Bermejo et al., 2016, 2018; Falace et al., 2021; Gálvez‐Pastor et al., 2025; García‐Gómez, Florido, Olaya‐Ponzone, Sempere‐Valverde, & Megina, 2021; Jueterbock et al., 2013; Riera et al., 2015).

Despite several records of decline and loss of *Cystoseira* s.l. forest across the Mediterranean, long‐term monitoring studies are remarkably scarce in its westernmost sector. This knowledge gap hinders effective conservation planning, particularly in critical transitional zones like the Strait of Gibraltar–Alboran Sea demarcation (ESAL, by its Spanish acronym). This region, one of the five official marine regions defined in the Spanish Marine Strategy under the EU Marine Strategy Framework Directive (Directive 2008/56/EC), is widely recognized as a biodiversity hotspot and a climatic refugium for both marine and terrestrial taxa (Coll et al., 2010; Flores‐Moya et al., 2021; Guerra‐García et al., 2009). Nevertheless, the ESAL demarcation is under increasing pressure from coastal development (De Andrés & Barragán Muñoz, 2022) and the proliferation of artificial substrata (Orfanidis et al., 2021) and, particularly, by the rapid spread of the invasive brown seaweed *Rugulopteryx okamurae* (Gálvez‐Pastor et al., 2025; García‐Gómez, Florido, Olaya‐Ponzone, Rey Díaz De Rada, et al., 2021; García‐Gómez, Florido, Olaya‐Ponzone, Sempere‐Valverde, & Megina, 2021; Román & Vázquez, 2025; Rosas‐Guerrero et al., 2025).

In this context, Species Distribution Models (SDMs) are powerful tools for identifying current and future habitats suitable for threatened species. By statistically relating known occurrence records to environmental and spatial predictors, SDMs provide spatially explicit forecasts useful for biodiversity conservation and climate‐change risk assessment (Booth et al., 2014; Frans et al., 2022; Srivastava et al., 2019). Among the available modeling approaches, Maximum Entropy Modeling (Maxent) is particularly suited for species with limited or fragmented distribution data (Hosseini et al., 2024; Phillips et al., 2017; Phillips & Dudík, 2008; Warren & Seifert, 2011), such as many seaweeds, and has been successfully applied to predict distribution shifts in species under changing environmental conditions (Battini et al., 2019; Martínez et al., 2018; Simon‐Nutbrown et al., 2020; Sudo et al., 2020).

Here, we applied SDMs to estimate the current and future potential distribution of two emblematic *Cystoseira* s.l. species within the ESAL region: *Gongolaria usneoides* and *Ericaria selaginoides*. These species represent contrasting yet ecologically significant components of the coastal benthic flora. Although *G. usneoides* typically inhabits the upper circalittoral and lower infralittoral zones, often co‐occurring with kelp and kelp‐like seaweeds (Flores‐Moya, 2012; Flores‐Moya et al., 2021), *E. selaginoides* dominates the upper sublittoral and plays a key structural role in shaping benthic assemblages (Bermejo et al., 2018; Flores‐Moya et al., 2021). The selection of these two species allows for a comparative analysis across different bathymetric zones and ecological roles. Specifically, this study aimed to: (1) identify the environmental drivers most likely to constrain the distribution of *G. usneoides* and *E. selaginoides* within the Strait–Alboran region, (2) quantify their current habitat suitability under present environmental conditions, and (3) project potential distributional shifts under future climate scenarios (SSP1‐1.9 and SSP5‐8.5, 2090–2100). The integration of driver identification, present‐day suitability, and future projections provides a spatially explicit basis for conservation planning and restoration prioritization under climate change.

## MATERIALS AND METHODS

### Occurrence data

Occurrence records for *Ericaria selaginoides* and *Gongolaria usneoides* were obtained exclusively from the Global Biodiversity Information Facility (GBIF.org, 2026; https://www.gbif.org) using the rgbif package in R. To ensure reproducibility, a formal GBIF occurrence download was generated using the GBIF download system, and the dataset is available under the following DOI: https:doi.org/10.15468/dl.h9x756. Records were restricted to the period 2000–2020 to match the temporal coverage of environmental baseline layers. All presence records were subjected to a standardized quality‐control protocol. We removed records lacking geographic coordinates, duplicated entries, records located on land, and those with obvious georeferencing errors. To reduce spatial clustering and sampling bias, occurrences were spatially thinned using a minimum nearest‐neighbor distance of 5 km, approximately matching the spatial resolution of the Bio‐ORACLE environmental layers (~0.05°). Records falling in cells with missing environmental values were excluded. As a result, the final number of occurrences used for model calibration was 93 occurrences for *E. selaginoides* and 15 for *G. usneoides*.

Addressing the recent taxonomic revision of *Cystoseira* s.l. by Molinari Novoa and Guiry (2020), the dataset was augmented by including the records corresponding to taxonomic synonyms within the same temporal window. Specifically, occurrences labeled as *Cystoseira usneoides* (Linnaeus) M. Roberts (a synonym for *Gongolaria usneoides*) and *C. tamariscifolia* (Hudson) Papenfuss (a synonym for *Ericaria selaginoides*) were incorporated. This taxonomic harmonization improved spatial coverage and reduced potential false absences arising from inconsistencies between historical and contemporary records (Figure 1).

**FIGURE 1:**
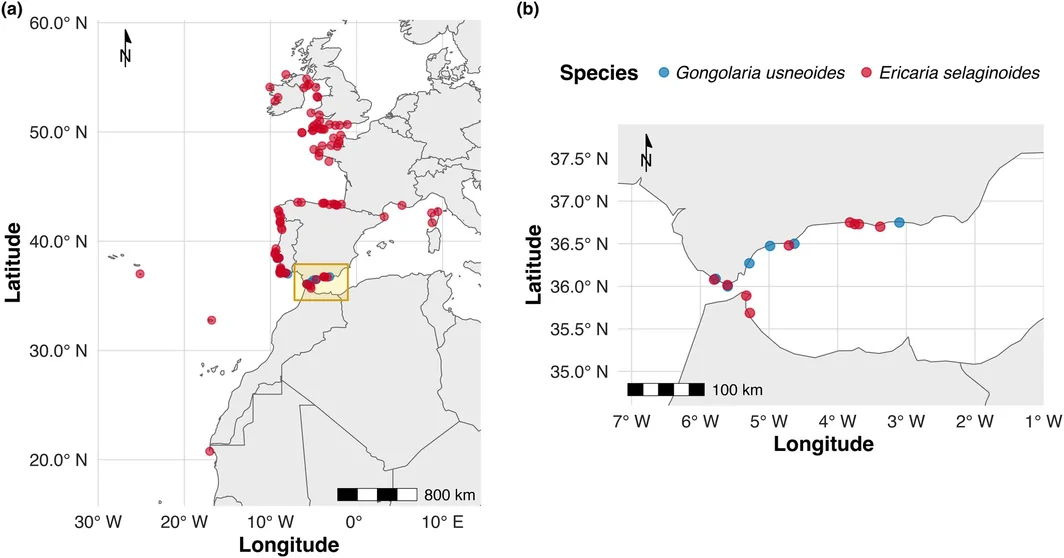
Occurrence records used for Maxent model calibration and visualization. (a) Thinned GBIF occurrence records (2000–2020) for *Gongolaria usneoides* and *Ericaria selaginoides* across the northeastern Atlantic–Mediterranean region; the shaded polygon indicates the Alboran Sea demarcation used as the focal study area for interpretation. (b) Zoomed view of the Alboran Sea showing the spatial distribution of the same occurrence records. MaxEnt baseline models were calibrated using all available thinned occurrence records for each species within the environmental calibration domain.

Dataset‐level provenance was retained by preserving the original GBIF datasetKey for each record. A complete list of contributing datasets, including publishers, is provided in Table S1. In addition, the full curated occurrence dataset used for model calibration is provided as Table S2.

### Environmental predictors

Environmental predictors were obtained from Bio‐ORACLE v3 (Assis et al., 2024), which provides globally harmonized marine environmental layers at 0.05° spatial resolution (~5–9 km). Bio‐ORACLE was originally developed by Tyberghein et al. (2012) and subsequently expanded (Assis et al., 2018).

A set of surface‐derived climatological predictors was initially considered to capture thermal, hydrodynamic, chemical, and productivity‐related dimensions of the marine environment. In addition to temperature and circulation variables, we included dissolved oxygen, chlorophyll *a* concentration, primary productivity, and mixed layer depth (MLD). These variables were incorporated as integrative proxies of broader physical and biogeochemical processes influencing benthic macroalgal habitats. For example, chlorophyll *a* reflects phytoplankton biomass and light attenuation in coastal waters (Cloern, 1996; Kirk, 2011), primary productivity integrates nutrient availability (Valiela et al., 1997), and MLD represents stratification and vertical mixing intensity (Behrenfeld & Boss, 2014; Sverdrup, 1953).

Collinearity among predictors was evaluated prior to model calibration. Pearson correlation coefficients were calculated using 10,000 randomly sampled grid cells within the calibration domain. Predictors with |r| > 0.7 were not retained simultaneously (Dormann et al., 2013). When collinearity occurred, variables were selected based on ecological relevance to seaweeds and completeness of spatial coverage. Nutrient proxies exhibiting strong redundancy were not included together. As a result, only a subset of the initially considered predictors was retained after correlation filtering. The final predictor set consisted exclusively of non‐redundant climatological mean variables available for both present and future projections (Figure S1; Table S3). A detailed description of the predictor collinearity assessment and variable selection procedure is provided in the Supplementary Methods (Appendix S1).

### Species distribution models

Species distribution models were fit using the MaxEnt algorithm (Phillips et al., 2006, 2017; Phillips & Dudík, 2008), implemented in R using the maxnet package (Phillips et al., 2017). Model tuning was conducted using ENMeval v2 (Muscarella et al., 2014), which was used to tune both feature classes and regularization multipliers. Linear (L), quadratic (Q), and hinge (H) features were allowed, whereas product (P) and threshold (T) features were excluded to reduce overfitting and improve ecological interpretability (Merow et al., 2013; Phillips & Dudík, 2008). Feature combinations were adapted to sample size. For *Gongolaria usneoides* (*n* = 15), only simpler feature sets (L, LQ) were evaluated. For *Ericaria selaginoides* (*n* = 93), more flexible combinations (L, LQ, H, LQH) were considered. Regularization multipliers were tuned across a predefined range, and final models were selected based on cross‐validated performance metrics. The optimal feature classes, regularization multipliers, and cross‐validation schemes selected for each species are summarized in Table S4.

Models were calibrated within species‐specific accessible areas (M) following the biotic‐abiotic‐mobility (BAM) framework (Barve et al., 2011; Soberon & Peterson, 2005). For each species, M was defined as the geographic bounding box of curated occurrences expanded by a 2° buffer in all directions. Environmental predictor layers were cropped to this extent prior to model calibration, ensuring that both presences and background points were sampled within the same accessible environmental domain. Background points *(n* = 10,000) were randomly sampled within M from the cropped predictor rasters. Model performance was assessed using spatially structured cross‐validation (Roberts et al., 2017). For *Ericaria selaginoides*, 4‐fold spatial block cross‐validation was applied, whereas for *Gongolaria usneoides*, leave‐one‐out jackknife cross‐validation was used due to the limited sample size (Pearson et al., 2007). Model discrimination was quantified using the area under the receiver operating characteristic curve (AUC), and omission rates at the 10th percentile training presence threshold (OR10) were also calculated. Present‐day projections were restricted to the same M extent, and future projections (SSP1‐1.9 and SSP5‐8.5, 2090–2100) were generated using environmental layers with identical spatial resolution and geographic extent to ensure comparability across time periods.

### Projection to future scenarios

Future projections were generated for SSP1‐1.9 and SSP5‐8.5 (2090–2100) using the calibrated models and corresponding future predictor layers. Environmental rasters were spatially aligned to ensure identical resolution and geographic extent across baseline and future conditions. Projections were restricted to the same geographic domain used for model calibration. Extrapolation beyond the environmental range of the calibration data was allowed, whereas clamping was disabled to avoid artificial truncation of suitability values at the limits of the training environmental space (Elith et al., 2011; Elith & Leathwick, 2009; Owens et al., 2013; Phillips et al., 2006).

### Computational environment

All analyses were conducted in R (R Core Team, 2024). Species distribution models were fit using the MaxEnt algorithm (Phillips et al., 2006) implemented through the maxnet engine within the ENMeval package, which allows automated tuning of model complexity and spatially independent model evaluation (Muscarella et al., 2014). Occurrence records were spatially thinned using the spThin package to reduce spatial sampling bias. Environmental raster processing was performed using terra and sf, while biodiversity occurrence data were retrieved using rgbif. Data manipulation and visualization were conducted using dplyr, ggplot2, viridis, ggspatial, and patchwork.

All scripts used for data processing, modeling, and figure generation are provided in the Supporting information.

## RESULTS

### Model performance

Species distribution models showed consistently high discriminatory ability under spatially structured cross‐validation. Mean AUC was 0.949 ± 0.02 for *Ericaria selaginoides* (4‐fold spatial block validation) and 0.982 ± 0.03 for *Gongolaria usneoides* (leave‐one‐out jackknife). The OR10 values were 0.122 for *E. selaginoides* and 0.133 for *G. usneoides*. Together, these metrics indicate stable model performance under spatially structured validation (Figure S2).

### Environmental drivers and niche structure

Permutation importance analyses identified mixed layer depth (mlotst_mean) as the dominant environmental driver for both species (Figure S2). In *Gongolaria usneoides*, the remaining predictors contributed only marginally to model discrimination, with minor secondary effects of silicate concentration and sea surface temperature. Similarly, in *Ericaria selaginoides*, all additional predictors showed comparatively low permutation importance, although sea surface temperature and cloud cover contributed weak secondary effects to model performance.

The limited contribution of most remaining predictors indicates that large‐scale vertical structure of the water column exerts stronger control over distribution patterns than horizontal chemical gradients. This pattern is consistent with the ecological requirements of canopy‐forming seaweeds, whose physiology and competitive performance are strongly mediated by stratification dynamics, nutrient renewal, and light penetration.

Low marginal effects in response curves should not be interpreted as ecological irrelevance. Supplementary analyses of environmental occupancy relative to background availability (Figure S3) indicate that several of the dominant predictors correspond to relatively restricted occupied environmental ranges within the M. For example, *Gongolaria usneoides* occupies a narrow fraction of the available mixed layer depth gradient, suggesting strong environmental filtering associated with stratification regimes.

### Present‐day distribution patterns

Under present‐day conditions, both species exhibited strongly coastal patterns of habitat suitability, consistent with their benthic nearshore ecology. *Ericaria selaginoides* showed its highest suitability concentrated along the northern margin of the Alboran Sea, particularly across the Atlantic‐influenced sectors of the southern Iberian Peninsula, with comparatively lower suitability across much of the southern margin. In contrast, *Gongolaria usneoides* displayed a more spatially fragmented pattern, with localized high‐suitability stretches primarily concentrated around the Strait of Gibraltar and adjacent western sectors, together with smaller suitable patches along the southern coastline. Overall, the comparative coastal projections highlight partial niche differentiation at the regional scale: *E. selaginoides* was associated with comparatively broader areas of favorable coastal habitat, whereas *G. usneoides* appeared restricted to a smaller number of environmentally suitable nearshore hotspots across the basin (Figure 2).

**FIGURE 2:**
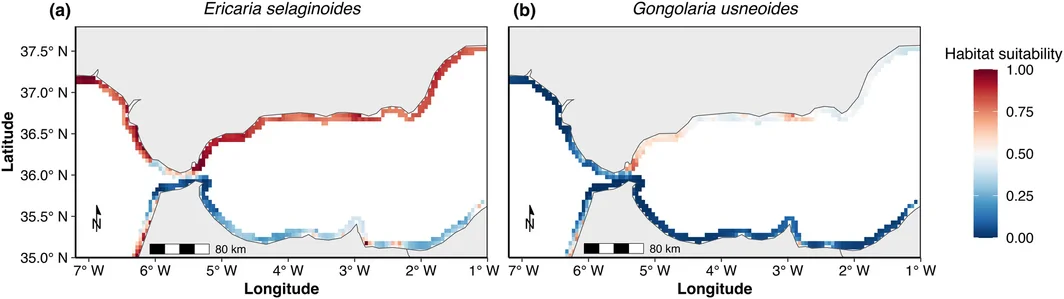
Present‐day coastal habitat suitability in the Alboran Sea. Habitat suitability derived from MaxEnt (cloglog output; 0–1) for *Ericaria selaginoides* (a) and *Gongolaria usneoides* (b) under baseline environmental conditions (Bio‐ORACLE climatology, 2000–2020). Predictions are shown only within a 10‐km coastal buffer to reflect the nearshore distribution of both taxa; offshore cells are masked. Higher values indicate higher habitat suitability.

### Future habitat shifts under climate scenarios

Projected responses differed markedly between species. *Ericaria selaginoides* exhibited mostly positive changes in habitat suitability under both SSP1‐1.9 and SSP5‐8.5, with gains distributed along extensive segments of the Alboran coastline (Figure 3a,c). Under the high‐emission scenario, positive changes became more continuous, suggesting a potential climatic expansion within the modeled coastal domain.

**FIGURE 3:**
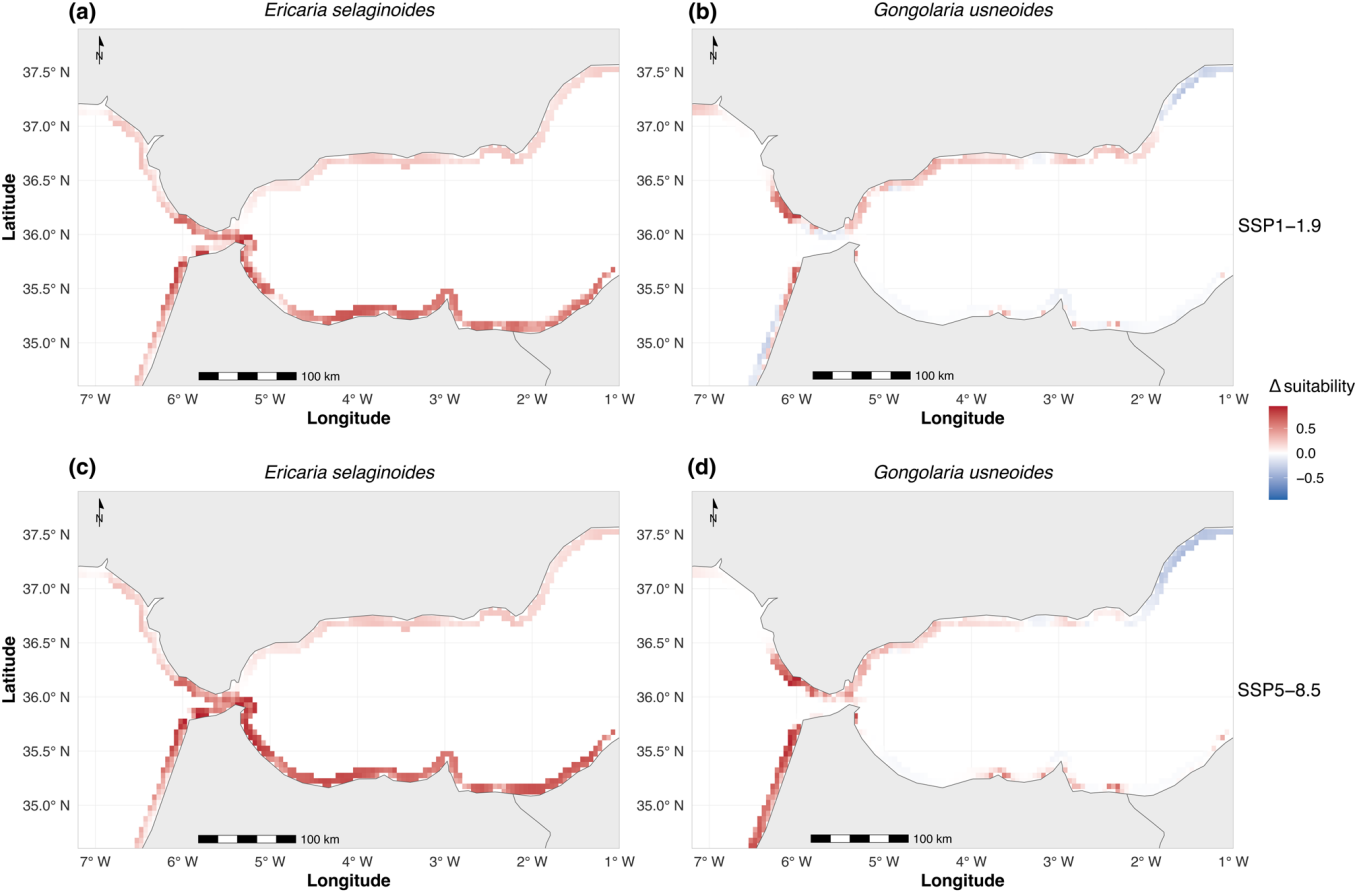
Changes in coastal habitat suitability (Δ suitability = Future − Present) for *Ericaria selaginoides* (a, c) and *Gongolaria usneoides* (b, d) along the Alboran Sea coastline, restricted to the ≤10 km coastal band. Panels a–b show changes under the low‐emission scenario SSP1‐1.9 (2090–2100), and panels c–d under the high‐emission scenario SSP5‐8.5 (2090–2100). Positive values indicate increased habitat suitability relative to the baseline period, whereas negative values indicate decreased suitability.

In contrast, *Gongolaria usneoides* showed spatially heterogeneous responses under both scenarios (Figure 3b,d). Under SSP1‐1.9, projected changes were generally weak, with localized suitability losses occurring mainly toward the eastern Alboran coastline and small suitability gains in western sectors. Under SSP5‐8.5, this spatial contrast intensified, with stronger suitability increases around the Strait of Gibraltar and the western Alboran Sea but persistent suitability losses across portions of the eastern coastline.

These projections indicate clear interspecific differences in climate sensitivity under future scenarios. Overall, *Gongolaria usneoides* showed a greater tendency toward suitability decline and fragmentation of climatically suitable habitat, whereas *Ericaria selaginoides* retained comparatively broader areas of stable or locally increased habitat suitability across the study region.

## DISCUSSION

Our results show that *Gongolaria usneoides* and *Ericaria selaginoides* occupy environmentally differentiated niches within the Alboran Sea and exhibit contrasting sensitivities to projected scenarios of climatic change in the coming decades. Although both species are canopy‐forming members of *Cystoseira* s.l., their modeled responses revealed distinct ecological niches. Mixed layer depth is a key predictor, highlighting the central role of vertical stratification in modulating light penetration, nutrient flux, and hydrodynamic stress in Mediterranean benthic systems (D'Ortenzio et al., 2005). However, the ecological interpretation of this driver differed between species, showing divergent environmental constraints.

*Gongolaria usneoides* was associated with a narrow portion of the available environmental gradient, suggesting strong environmental filtering and limited tolerance to shifts in stratification or associated hydrographic conditions. In contrast, *Ericaria selaginoides* exhibited broader tolerance across physical gradients but detectable secondary sensitivity to biogeochemical proxies, particularly those related to productivity and nutrient structure. This distinction is consistent with previous evidence that Mediterranean fucoids respond not only to irradiance and temperature but also to trophic regime stability and nutrient balance (Celis‐Plá et al., 2014, 2017). Thus, despite phylogenetic proximity, the two species appear to occupy partially decoupled niche dimensions.

Projected future shifts revealed clear interspecific differences in climate responses. Under SSP1‐1.9, changes were moderate but directionally consistent. *Ericaria selaginoides* retained suitability across portions of its current coastal range and exhibited localized increases in some sectors together with declines in others, suggesting comparatively broader environmental tolerance under limited warming. In contrast, *Gongolaria usneoides* showed suitability reductions even under this low‐emission scenario, indicating greater sensitivity to relatively modest environmental shifts.

Under SSP5‐8.5, responses diverged between species more strongly. *Gongolaria usneoides* showed a predominance of declines in suitability across large portions of the Alboran coastline, particularly toward eastern sectors, although localized suitability increases persisted in some western areas around the Strait of Gibraltar. In contrast, *Ericaria selaginoides* displayed spatially heterogeneous responses, with predominantly positive changes in habitat suitability, although localized declines persisted in some sectors. These patterns likely reflect projected hydrographic restructuring that locally aligns with its broader environmental tolerance. However, such modeled increases should be interpreted cautiously: Suitability gains do not necessarily imply demographic expansion or realized colonization, particularly in fragmented coastal systems where dispersal and connectivity constraints may limit the occupation of newly suitable habitats (Assis et al., 2026).

The two future scenarios produced partially convergent spatial outcomes. This convergence does not necessarily imply climate insensitivity. Rather, it may reflect the fact that dominant predictors in the models (especially mixed layer depth and related hydrographic structure) are not purely temperature driven but integrate stratification dynamics and nutrient mixing processes. At the regional scale of the Alboran Sea, projected changes in these structural variables may be directionally similar under both SSP1‐1.9 and SSP5‐8.5, even if warming intensity differs. Moreover, for environmentally restricted taxa such as *Gongolaria usneoides*, even moderate environmental shifts under a low‐emission pathway may exceed tolerance limits, resulting in contraction under both scenarios. In this line of reasoning, the exclusion of certain light‐related predictors from future projections may reduce sensitivity to radiative differences between scenarios. Together, these factors suggest that apparent scenario convergence may arise from threshold responses to stratification and hydrographic restructuring rather than from a lack of climatic forcing per se.

The differential projections between species are ecologically significant. Canopy‐forming fucoids function as ecosystem engineers, structuring biodiversity and modulating trophic networks (Mancuso et al., 2018, 2021). A decline of *Gongolaria usneoides* could alter canopy architecture and community composition, potentially shifting benthic assemblages toward structurally simplified states. Conversely, the projected persistence and local gains of *Ericaria selaginoides* under certain scenarios suggest that species‐specific responses may buffer but not eliminate broader functional change.

These projections are consistent with the documented sensitivity of Mediterranean fucoids to climatic forcing (Faga & Gurgel, 2024; Falace et al., 2021; Neiva et al., 2016). Intensified stratification and warming in the Mediterranean basin are expected to reduce vertical nutrient mixing and alter thermal regimes (Intergovernmental Panel on Climate Change, 2021), potentially narrowing environmental windows for species dependent on stable hydrographic conditions. The stronger vulnerability of *Gongolaria usneoides* suggests that environmentally restricted taxa may face disproportionate risk under ongoing climatic change. The weak or near‐flat marginal responses observed for several predictors do not necessarily indicate a lack of ecological relevance; rather they suggest that these variables do not exert strong independent effects within the multivariate environmental space. Instead, their influence may be indirect, mediated through correlations with dominant drivers such as mixed layer depth, or expressed only through interactions not captured by marginal response curves (Figure S2).

Several caveats warrant careful consideration. First, the model for *Gongolaria usneoides* is based on a limited number of occurrences, which increases uncertainty in response estimation and may increase discrimination metrics despite the use of jackknife cross‐validation and complexity control (Pearson et al., 2007; Warren & Seifert, 2011). Consequently, projections for *G. usneoides* should be interpreted with appropriate caution, particularly when transferred beyond the range of conditions represented in the calibration data. Second, occurrence data were derived from GBIF records, which are known to exhibit spatial sampling bias toward well‐surveyed coastal areas (Beck et al., 2014; Meyer et al., 2016; Ruete, 2015). Spatial thinning and block cross‐validation reduce, but do not fully eliminate, this bias. More broadly, model outputs represent habitat suitability rather than true occurrence probability (Elith & Leathwick, 2009) and do not incorporate dispersal constraints, demographic processes, or biotic interactions.

Projections further assume static species–environment interactions and do not account for physiological acclimation, adaptive evolution, or shifts in competitive dynamics under future conditions. In addition, correlative approaches such as MaxEnt may not fully capture the complete environmental niche of a species, particularly when realized distributions underestimate physiological tolerances or reflect non‐equilibrium distributions (Laeseke et al., 2021, 2024). Consequently, projected suitability patterns should be interpreted as estimates of potential environmental association rather than direct representations of the full physiological niche. Although models were calibrated within species‐specific accessible areas following the BAM framework, uncertainty remains regarding fine‐scale dispersal barriers, coastal geomorphology, and microhabitat heterogeneity that are not resolved at the 0.05° spatial scale.

Additionally, future projections were constrained to environmental predictors available under SSP scenarios. Although this ensured consistency between baseline and future models, the exclusion of certain light‐related variables (e.g., photosynthetically active radiation) may limit the representation of key physiological drivers of canopy‐forming macroalgae. Finally, the rapid expansion of the invasive alga *Rugulopteryx okamurae* along southern Iberian coasts introduces an additional biotic stressor not incorporated into the models (Borriglione et al., 2024; García‐Gómez, Florido, Olaya‐Ponzone, Rey Díaz De Rada, et al., 2021; García‐Gómez, Florido, Olaya‐Ponzone, Sempere‐Valverde, & Megina, 2021; Laamraoui et al., 2024; Rueda et al., 2023). Interactions between climate‐driven environmental shifts and biological invasions may substantially alter realized distributions beyond what abiotic suitability alone predicts.

From a biodiversity conservation perspective, the spatially explicit projections generated in this study provide a valuable framework for guiding restoration and conservation planning of Mediterranean fucoid forests. Areas of persistent or increasing habitat suitability for *Ericaria selaginoides* may represent priority targets for active restoration, particularly where local stressors are manageable and substrate availability is not limiting. In contrast, the projected contraction of suitable habitat for *Gongolaria usneoides* suggests that restoration efforts for this species should be approached cautiously and focused on locations where environmental conditions remain within its narrower tolerance range.

Future work should integrate mechanistic demographic models, incorporate dispersal kernels, and explicitly evaluate interactions with invasive species and local stressors. Coupling species distribution models with high‐resolution hydrodynamic simulations and long‐term monitoring would improve understanding of how stratification dynamics translate into benthic ecological outcomes. Experimental assessments of thermal and nutrient tolerance thresholds would further refine predictive capacity.

## AUTHOR CONTRIBUTIONS

**Pablo Pérez‐Castro:** Conceptualization (equal); data curation (equal); formal analysis (equal); visualization (equal); writing – original draft (equal); writing – review and editing (supporting). **Elena Bañares‐España:** Conceptualization (equal); funding acquisition (equal); methodology (equal); visualization (equal); writing – review and editing (equal). **Antonio Flores‐Moya:** Conceptualization (equal); formal analysis (equal); funding acquisition (lead); methodology (equal); supervision (equal); writing – review and editing (equal). **Ignacio J. Melero‐Jiménez:** Conceptualization (lead); data curation (lead); formal analysis (lead); funding acquisition (supporting); investigation (lead); methodology (lead); supervision (lead); visualization (equal); writing – original draft (lead); writing – review and editing (lead).

## Supporting information


**Figure S1.** Pairwise Pearson correlation matrix (|r|) among candidate environmental predictors.
**Figure S2.** Model performance and variable importance for *Ericaria selaginoides* and *Gongolaria*.
**Figure S3.** Environmental gradient occupancy within the accessible area (M) for *Ericaria selaginoides*.
**Table S4.** Model calibration settings and optimal parameters selected during ENMeval tuning. The number of presences corresponds to the final dataset used for model calibration after spatial thinning. Models were selected using a hierarchical criterion prioritizing minimum omission rate at the 10th percentile training presence threshold (OR10) and, secondarily, maximum validation AUC.

**Table S1.** GBIF datasets contributing occurrence records for *Ericaria selaginoides* and *Gongolaria usneoides* used in model calibration after spatial thinning.

**Table S2.** Geographic coordinates and metadata of occurrence records used to calibrate the species distribution models after data cleaning and 5‐km spatial thinning.

**Table S3.** Bio‐ORACLE environmental datasets retained after correlation filtering and considered as candidate predictors for present and future species distribution modeling.

**Data S1:** Supporting Information.

## Data Availability

All data, supplementary materials, and R code supporting the findings of this study are available in the Supporting Information. Occurrence records were obtained from the Global Biodiversity Information Facility (GBIF) and are available through the download DOI: 10.15468/dl.h9x756. The R workflow enables full reproduction of the analyses, including data retrieval, environmental data processing, species distribution model calibration, future climate projections, and figure generation.

## References

[jpy70209-bib-0001] Álvarez‐Canali, D. , Sangil, C. , Reyes, J. , & Sansón, M. (2019). Local variations in environmental parameters govern 50 years of the decline of *Fucus guiryi* populations in the Canary Islands (eastern Atlantic). Journal of Sea Research, 155, 101823. 10.1016/j.seares.2019.101823

[jpy70209-bib-0002] Assis, J. , Fernández Bejarano, S. J. , Salazar, V. W. , Schepers, L. , Gouvêa, L. , Fragkopoulou, E. , Leclercq, F. , Vanhoorne, B. , Tyberghein, L. , Serrão, E. A. , Verbruggen, H. , & De Clerck, O. (2024). bio‐oracle v3.0. Pushing marine data layers to the cmip6 earth system models of climate change research. Global Ecology and Biogeography, 33(4), e13813. 10.1111/geb.13813

[jpy70209-bib-0003] Assis, J. , Fragkopoulou, E. , Serrão, E. A. , & Araújo, M. B. (2026). Oceanographic connectivity strongly restricts future range expansions of critical marine forest species. npj Biodiversity, 5(1), 10. 10.1038/s44185-026-00123-y PMC13031816 41896310

[jpy70209-bib-0004] Assis, J. , Tyberghein, L. , Bosch, S. , Verbruggen, H. , Serrão, E. A. , & De Clerck, O. (2018). Bio‐ORACLE v2.0: Extending marine data layers for bioclimatic modelling. Global Ecology and Biogeography, 27(3), 277–284. 10.1111/geb.12693

[jpy70209-bib-0005] Báez, J. C. , Olivero, J. , Real, R. , Vargas, J. M. , & Flores‐Moya, A. (2005). Analysis of geographical variation in species richness within the genera *Audouinella* (Rhodophyta), *Cystoseira* (Phaeophyceae) and *Cladophora* (Chlorophyta) in the western Mediterranean Sea. Botanica Marina, 48(1), 30–37. 10.1515/BOT.2005.012

[jpy70209-bib-0006] Báez, J. C. , Real, R. , Vargas, J. M. , & Flores‐Moya, A. (2004). A biogeographical analysis of the genera *Audouinella* (Rhodophyta), *Cystoseira* (Phaeophyceae) and *Cladophora* (Chlorophyta) in the western Mediterranean Sea and Adriatic Sea. Phycologia, 43(4), 404–405. 10.2216/i0031-8884-43-4-404.1

[jpy70209-bib-0007] Báez, J. C. , Real, R. , Vargas, J. M. , & Flores‐Moya, A. (2005). Chorotypes of seaweeds from the western Mediterranean Sea and the Adriatic Sea: An analysis based on the genera *Audouinella* (Rhodophyta), *Cystoseira* (Phaeophyceae) and *Cladophora* (Chlorophyta). Phycological Research, 53(4), 255–265. 10.1111/j.1440-183.2005.00393.x

[jpy70209-bib-0008] Ballesteros, E. (1990). Structure and dynamics of the community of *Cystoseira zosteroides* (Turner) C. Agardh (Fucales, Phaeophyceae) in the northwestern Mediterranean. Scientia Marina, 54, 217–229.

[jpy70209-bib-0009] Barve, N. , Barve, V. , Jiménez‐Valverde, A. , Lira‐Noriega, A. , Maher, S. P. , Peterson, A. T. , Soberón, J. , & Villalobos, F. (2011). The crucial role of the accessible area in ecological niche modeling and species distribution modeling. Ecological Modelling, 222(11), 1810–1819. 10.1016/j.ecolmodel.2011.02.011

[jpy70209-bib-0010] Battini, N. , Farías, N. , Giachetti, C. , Schwindt, E. , & Bortolus, A. (2019). Staying ahead of invaders: Using species distribution modeling to predict alien species' potential niche shifts. Marine Ecology Progress Series, 612, 127–140. 10.3354/meps12878

[jpy70209-bib-0011] Beck, J. , Böller, M. , Erhardt, A. , & Schwanghart, W. (2014). Spatial bias in the GBIF database and its effect on modeling species' geographic distributions. Ecological Informatics, 19, 10–15. 10.1016/j.ecoinf.2013.11.002

[jpy70209-bib-0012] Behrenfeld, M. J. , & Boss, E. S. (2014). Resurrecting the ecological underpinnings of ocean plankton blooms. Annual Review of Marine Science, 6(1), 167–194. 10.1146/annurev-marine-052913-021325 24079309

[jpy70209-bib-0013] Bermejo, R. , Chefaoui, R. M. , Engelen, A. H. , Buonomo, R. , Neiva, J. , Ferreira‐Costa, J. , Pearson, G. A. , Marbà, N. , Duarte, C. M. , Airoldi, L. , Hernández, I. , Guiry, M. D. , & Serrão, E. A. (2018). Marine forests of the Mediterranean‐Atlantic *Cystoseira tamariscifolia* complex show a southern Iberian genetic hotspot and no reproductive isolation in parapatry. Scientific Reports, 8(1), 10427. 10.1038/s41598-018-28811-1 PMC6041324 29993019

[jpy70209-bib-0014] Bermejo, R. , De La Fuente, G. , Ramírez‐Romero, E. , Vergara, J. J. , & Hernández, I. (2016). Spatial variability and response to anthropogenic pressures of assemblages dominated by a habitat forming seaweed sensitive to pollution (northern coast of Alboran Sea). Marine Pollution Bulletin, 105(1), 255–264. 10.1016/j.marpolbul.2016.02.017 26892204

[jpy70209-bib-0015] Blanfuné, A. , Boudouresque, C.‐F. , Verlaque, M. , & Thibaut, T. (2025). Severe decline of *Gongolaria barbata* (Fucales) along most of the French Mediterranean coast. Scientific Reports, 15(1), 5701. 10.1038/s41598-025-89958-2 PMC11832745 39962211

[jpy70209-bib-0016] Booth, T. H. , Nix, H. A. , Busby, J. R. , & Hutchinson, M. F. (2014). bioclim: The first species distribution modelling package, its early applications and relevance to most current maxent studies. Diversity and Distributions, 20(1), 1–9. 10.1111/ddi.12144

[jpy70209-bib-0017] Borriglione, M. , Ruitton, S. , Boyer, F. , Thibault, D. , Blanfuné, A. , Guillemain, D. , Verlaque, M. , Boudouresque, C.‐F. , & Thibaut, T. (2024). Impact of the invasive brown alga *Rugulopteryx okamurae* on the benthic communities in the northwestern Mediterranean Sea. Estuarine, Coastal and Shelf Science, 310, 109010. 10.1016/j.ecss.2024.109010

[jpy70209-bib-0018] Bruno De Sousa, C. , Cox, C. J. , Brito, L. , Pavão, M. M. , Pereira, H. , Ferreira, A. , Ginja, C. , Campino, L. , Bermejo, R. , Parente, M. , & Varela, J. (2019). Improved phylogeny of brown algae *Cystoseira* (Fucales) from the Atlantic‐Mediterranean region based on mitochondrial sequences. PLoS ONE, 14(1), e0210143. 10.1371/journal.pone.0210143 PMC6364706 30699208

[jpy70209-bib-0019] Celis‐Plá, P. , Martínez, B. , Quintano, E. , García‐Sánchez, M. , Pedersen, A. , Navarro, N. , Copertino, M. , Mangaiyarkarasi, N. , Mariath, R. , Figueroa, F. , & Korbee, N. (2014). Short‐term ecophysiological and biochemical responses of *Cystoseira tamariscifolia* and *Ellisolandia elongata* to environmental changes. Aquatic Biology, 22, 227–243. 10.3354/ab00573

[jpy70209-bib-0020] Celis‐Plá, P. S. M. , Martínez, B. , Korbee, N. , Hall‐Spencer, J. M. , & Figueroa, F. L. (2017). Ecophysiological responses to elevated CO2 and temperature in *Cystoseira tamariscifolia* (Phaeophyceae). Climatic Change, 142(1–2), 67–81. 10.1007/s10584-017-1943-y

[jpy70209-bib-0021] Clausing, R. J. , Falace, A. , De La Fuente, G. , Della Torre, C. , Chiantore, M. , & Asnaghi, V. (2024). *Ex‐situ* restoration of the Mediterranean forest‐forming macroalga *Ericaria amentacea*: Optimizing growth in culture may not be the key to growth in the field. Marine Environmental Research, 202, 106718. 10.1016/j.marenvres.2024.106718 39232470

[jpy70209-bib-0022] Cloern, J. E. (1996). Phytoplankton bloom dynamics in coastal ecosystems: A review with some general lessons from sustained investigation of San Francisco Bay, California. Reviews of Geophysics, 34(2), 127–168. 10.1029/96RG00986

[jpy70209-bib-0023] Coll, M. , Piroddi, C. , Steenbeek, J. , Kaschner, K. , Ben Rais Lasram, F. , Aguzzi, J. , Ballesteros, E. , Bianchi, C. N. , Corbera, J. , Dailianis, T. , Danovaro, R. , Estrada, M. , Froglia, C. , Galil, B. S. , Gasol, J. M. , Gertwagen, R. , Gil, J. , Guilhaumon, F. , Kesner‐Reyes, K. , & Voultsiadou, E. (2010). The biodiversity of the Mediterranean Sea: Estimates, patterns, and threats. PLoS ONE, 5(8), e11842. 10.1371/journal.pone.0011842 PMC2914016 20689844

[jpy70209-bib-0024] De Andrés, M. , & Barragán Muñoz, J. M. (2022). The limits of coastal and marine areas in Andalusia (Spain). A socio‐ecological approach for ecosystem‐based management. Land Use Policy, 120, 106250. 10.1016/j.landusepol.2022.106250

[jpy70209-bib-0025] Dormann, C. F. , Elith, J. , Bacher, S. , Buchmann, C. , Carl, G. , Carré, G. , Marquéz, J. R. G. , Gruber, B. , Lafourcade, B. , Leitão, P. J. , Münkemüller, T. , McClean, C. , Osborne, P. E. , Reineking, B. , Schröder, B. , Skidmore, A. K. , Zurell, D. , & Lautenbach, S. (2013). Collinearity: A review of methods to deal with it and a simulation study evaluating their performance. Ecography, 36(1), 27–46. 10.1111/j.1600-0587.2012.07348.x

[jpy70209-bib-0026] D'Ortenzio, F. , Iudicone, D. , De Boyer Montegut, C. , Testor, P. , Antoine, D. , Marullo, S. , Santoleri, R. , & Madec, G. (2005). Seasonal variability of the mixed layer depth in the Mediterranean Sea as derived from in situ profiles. Geophysical Research Letters, 32(12), 2005GL022463. 10.1029/2005GL022463

[jpy70209-bib-0027] Elith, J. , & Leathwick, J. R. (2009). Species distribution models: Ecological explanation and prediction across space and time. Annual Review of Ecology, Evolution, and Systematics, 40(1), 677–697. 10.1146/annurev.ecolsys.110308.120159

[jpy70209-bib-0028] Elith, J. , Phillips, S. J. , Hastie, T. , Dudík, M. , Chee, Y. E. , & Yates, C. J. (2011). A statistical explanation of MaxEnt for ecologists: Statistical explanation of MaxEnt. Diversity and Distributions, 17(1), 43–57. 10.1111/j.1472-4642.2010.00725.x

[jpy70209-bib-0029] Faga, F. , & Gurgel, C. F. D. (2024). Distributional range shifts of Western Atlantic benthic *sargassum* species (Fucales, Phaeophyceae) under future climate change scenarios. Aquatic Botany, 190, 103705. 10.1016/j.aquabot.2023.103705

[jpy70209-bib-0030] Falace, A. , Kaleb, S. , De La Fuente, G. , Asnaghi, V. , & Chiantore, M. (2018). *Ex situ* cultivation protocol for *Cystoseira amentacea* var. *stricta* (Fucales, Phaeophyceae) from a restoration perspective. PLoS ONE, 13(2), e0193011. 10.1371/journal.pone.0193011 PMC5813978 29447238

[jpy70209-bib-0031] Falace, A. , Marletta, G. , Savonitto, G. , Candotto Carniel, F. , Srijemsi, M. , Bevilacqua, S. , Tretiach, M. , & Alongi, G. (2021). Is the South‐Mediterranean canopy‐forming *Ericaria giacconei* (= *Cystoseira hyblaea*) a loser from ocean warming? Frontiers in Marine Science, 8, 760637. 10.3389/fmars.2021.760637

[jpy70209-bib-0032] Flores‐Moya, A. (2012). Warm temperate seaweed communities: A case study of deep water kelp forests from the Alboran Sea (SW Mediterranean Sea) and the strait of Gibraltar. In C. Wiencke & K. Bischof (Eds.), Seaweed biology (Vol. 219, pp. 315–327). Springer Berlin Heidelberg. 10.1007/978-3-642-28451-9_15

[jpy70209-bib-0033] Flores‐Moya, A. , Moreno, D. , De La Rosa, J. , Altamirano, M. , & Bañares‐España, E. (2021). Seaweeds and seagrasses: The marine forests from the Alboran Sea. In J. C. Báez , J.‐T. Vázquez , J. A. Camiñas , & M. Malouli Idrissi (Eds.), Alboran Sea—Ecosystems and marine resources (pp. 247–284). Springer International Publishing. 10.1007/978-3-030-65516-7_8

[jpy70209-bib-0034] Frans, V. F. , Augé, A. A. , Fyfe, J. , Zhang, Y. , McNally, N. , Edelhoff, H. , Balkenhol, N. , & Engler, J. O. (2022). Integrated SDM database: Enhancing the relevance and utility of species distribution models in conservation management. Methods in Ecology and Evolution, 13(1), 243–261. 10.1111/2041-210X.13736

[jpy70209-bib-0035] Gálvez‐Pastor, E. , Sánchez De Pedro, R. , García‐Sánchez, M. J. , Flores‐Moya, A. , & Bañares‐España, E. (2025). *In vitro* negative effects of beach‐cast invasive marine seaweed *Rugulopteryx okamurae* across life‐stages of a native foundational species. Marine Environmental Research, 208, 107105. 10.1016/j.marenvres.2025.107105 40199063

[jpy70209-bib-0036] García‐Gómez, J. C. , Florido, M. , Olaya‐Ponzone, L. , Rey Díaz De Rada, J. , Donázar‐Aramendía, I. , Chacón, M. , Quintero, J. J. , Magariño, S. , & Megina, C. (2021). Monitoring extreme impacts of *Rugulopteryx okamurae* (Dictyotales, Ochrophyta) in El Estrecho Natural Park (biosphere reserve). Showing radical changes in the underwater seascape. Frontiers in Ecology and Evolution, 9, 639161. 10.3389/fevo.2021.639161

[jpy70209-bib-0037] García‐Gómez, J. C. , Florido, M. , Olaya‐Ponzone, L. , Sempere‐Valverde, J. , & Megina, C. (2021). The invasive macroalga *Rugulopteryx okamurae*: Substrata plasticity and spatial colonization pressure on resident macroalgae. Frontiers in Ecology and Evolution, 9, 631754. 10.3389/fevo.2021.631754

[jpy70209-bib-0038] GBIF.org . (2026). GBIF Occurrence Download. 10.15468/dl.h9x756

[jpy70209-bib-0039] Guerra‐García, J. M. , Cabezas, P. , Baeza‐Rojano, E. , Espinosa, F. , & García‐Gómez, J. C. (2009). Is the north side of the strait of Gibraltar more diverse than the south side? A case study using the intertidal peracarids (Crustacea: Malacostraca) associated to the seaweed *Corallina elongata* . Journal of the Marine Biological Association of the United Kingdom, 89(2), 387–397. 10.1017/S0025315409002938

[jpy70209-bib-0084] Habitats Directive (92/43/EEC) . (1992). Council Directive 92/43/EEC of 21 May 1992 on the conservation of natural habitats and of wild fauna and flora. Official Journal of the European Communities, L206, 7–50. https://eur‐lex.europa.eu/eli/dir/1992/43/oj

[jpy70209-bib-0040] Hereu, B. , Mangialajo, L. , Ballesteros, E. , & Thibaut, T. (2008). On the occurrence, structure and distribution of deep‐water *Cystoseira* (Phaeophyceae) populations in the Port‐Cros National Park (north‐western Mediterranean). European Journal of Phycology, 43(3), 263–273. 10.1080/09670260801930330

[jpy70209-bib-0041] Hosseini, N. , Ghorbanpour, M. , & Mostafavi, H. (2024). Habitat potential modelling and the effect of climate change on the current and future distribution of three *Thymus* species in Iran using MaxEnt. Scientific Reports, 14(1), 3641. 10.1038/s41598-024-53405-5 PMC10864348 38351276

[jpy70209-bib-0042] Intergovernmental Panel on Climate Change . (2021). Climate change 2021: The physical science basis. Contribution of working group I to the sixth assessment report of the intergovernmental panel on climate change. Cambridge University Press. 10.1017/9781009157896

[jpy70209-bib-0043] Iveša, L. , Djakovac, T. , & Devescovi, M. (2016). Long‐term fluctuations in *Cystoseira* populations along the west Istrian coast (Croatia) related to eutrophication patterns in the northern Adriatic Sea. Marine Pollution Bulletin, 106(1–2), 162–173. 10.1016/j.marpolbul.2016.03.010 26975612

[jpy70209-bib-0044] Jueterbock, A. , Tyberghein, L. , Verbruggen, H. , Coyer, J. A. , Olsen, J. L. , & Hoarau, G. (2013). Climate change impact on seaweed meadow distribution in the North Atlantic rocky intertidal. Ecology and Evolution, 3(5), 1356–1373. 10.1002/ece3.541 PMC3678489 23762521

[jpy70209-bib-0045] Kirk, J. T. O. (2011). Light and photosynthesis in aquatic ecosystems (3rd ed.). Cambridge University Press.

[jpy70209-bib-0046] Laamraoui, M. R. , Mghili, B. , Roca, M. , Chaieb, O. , Ostalé‐Valriberas, E. , Martín‐Zorrillae, A. , Sabino‐Lorenzo, A. , & Aarab, S. (2024). Rapid invasion and expansion of the invasive macroalgae *Rugulopteryx okamurae* in the Mediterranean and Atlantic: A 10‐year review. Marine Pollution Bulletin, 209, 117194. 10.1016/j.marpolbul.2024.117194 39476635

[jpy70209-bib-0047] Laeseke, P. , Martínez, B. , Mansilla, A. , & Bischof, K. (2021). Future range dynamics of the red alga *Capreolia implexa* in native and invaded regions: Contrasting predictions from species distribution models versus physiological knowledge. Biological Invasions, 23, 3279–3280. 10.1007/s10530-021-02582-9

[jpy70209-bib-0048] Laeseke, P. , Martínez, B. D.‐C. , & Bischof, K. (2024). Large‐scale deviations between realized and fundamental thermal niches in global seaweed distributions. Diversity and Distributions, 30, e13868. 10.1111/ddi.13868

[jpy70209-bib-0049] Mancuso, F. P. , Milazzo, M. , & Chemello, R. (2021). Decreasing in patch‐size of *Cystoseira* forests reduces the diversity of their associated molluscan assemblage in Mediterranean rocky reefs. Estuarine, Coastal and Shelf Science, 250, 107163. 10.1016/j.ecss.2020.107163

[jpy70209-bib-0050] Mancuso, F. P. , Strain, E. M. A. , Piccioni, E. , De Clerck, O. , Sarà, G. , & Airoldi, L. (2018). Status of vulnerable *Cystoseira* populations along the Italian infralittoral fringe, and relationships with environmental and anthropogenic variables. Marine Pollution Bulletin, 129(2), 762–771. 10.1016/j.marpolbul.2017.10.068 29108739

[jpy70209-bib-0051] Martínez, B. , Radford, B. , Thomsen, M. S. , Connell, S. D. , Carreño, F. , Bradshaw, C. J. A. , Fordham, D. A. , Russell, B. D. , Gurgel, C. F. D. , & Wernberg, T. (2018). Distribution models predict large contractions of habitat‐forming seaweeds in response to ocean warming. Diversity and Distributions, 24(10), 1350–1366. 10.1111/ddi.12767

[jpy70209-bib-0052] Merow, C. , Smith, M. J. , & Silander, J. A. (2013). A practical guide to MaxEnt for modeling species' distributions: What it does, and why inputs and settings matter. Ecography, 36(10), 1058–1069. 10.1111/j.1600-0587.2013.07872.x

[jpy70209-bib-0053] Meyer, C. , Weigelt, P. , & Kreft, H. (2016). Multidimensional biases, gaps and uncertainties in global plant occurrence information. Ecology Letters, 19(8), 992–1006. 10.1111/ele.12624 27250865

[jpy70209-bib-0083] Molinari Novoa, E. , & Guiry, M. D. (2020). Reinstatement of the genera Gongolaria Boehmer and Ericaria Stackhouse (Sargassaceae, Phaeophyceae). Notulae Algarum, 172, 1–10.

[jpy70209-bib-0054] Muscarella, R. , Galante, P. J. , Soley‐Guardia, M. , Boria, R. A. , Kass, J. M. , Uriarte, M. , & Anderson, R. P. (2014). enm eval: An R package for conducting spatially independent evaluations and estimating optimal model complexity for maxent ecological niche models. Methods in Ecology and Evolution, 5(11), 1198–1205. 10.1111/2041-210X.12261

[jpy70209-bib-0055] Neiva, J. , Serrão, E. A. , Assis, J. , Pearson, G. A. , Coyer, J. A. , Olsen, J. L. , Hoarau, G. , & Valero, M. (2016). Climate oscillations, range shifts and phylogeographic patterns of North Atlantic Fucaceae. In Z.‐M. Hu & C. Fraser (Eds.), Seaweed Phylogeography (pp. 279–308). Springer. 10.1007/978-94-017-7534-2_11

[jpy70209-bib-0056] Orellana, S. , Hernández, M. , & Sansón, M. (2019). Diversity of *Cystoseira sensu lato* (Fucales, Phaeophyceae) in the eastern Atlantic and Mediterranean based on morphological and DNA evidence, including *Carpodesmia* gen. emend. and *Treptacantha* gen. emend. European Journal of Phycology, 54(3), 447–465. 10.1080/09670262.2019.1590862

[jpy70209-bib-0057] Orfanidis, S. , Rindi, F. , Cebrian, E. , Fraschetti, S. , Nasto, I. , Taskin, E. , Bianchelli, S. , Papathanasiou, V. , Kosmidou, M. , Caragnano, A. , Tsioli, S. , Ratti, S. , Fabbrizzi, E. , Verdura, J. , Tamburello, L. , Beqiraj, S. , Kashta, L. , Sota, D. , Papadimitriou, A. , & Danovaro, R. (2021). Effects of natural and anthropogenic stressors on Fucalean brown seaweeds across different spatial scales in the Mediterranean Sea. Frontiers in Marine Science, 8, 658417. 10.3389/fmars.2021.658417

[jpy70209-bib-0058] Owens, H. L. , Campbell, L. P. , Dornak, L. L. , Saupe, E. E. , Barve, N. , Soberón, J. , Ingenloff, K. , Lira‐Noriega, A. , Hensz, C. M. , Myers, C. E. , & Peterson, A. T. (2013). Constraints on interpretation of ecological niche models by limited environmental ranges on calibration areas. Ecological Modelling, 263, 10–18. 10.1016/j.ecolmodel.2013.04.011

[jpy70209-bib-0059] Pearson, R. G. , Raxworthy, C. J. , Nakamura, M. , & Townsend Peterson, A. (2007). Predicting species distributions from small numbers of occurrence records: A test case using cryptic geckos in Madagascar. Journal of Biogeography, 34(1), 102–117. 10.1111/j.1365-2699.2006.01594.x

[jpy70209-bib-0060] Phillips, S. J. , Anderson, R. P. , Dudík, M. , Schapire, R. E. , & Blair, M. E. (2017). Opening the black box: An open‐source release of Maxent. Ecography, 40(7), 887–893. 10.1111/ecog.03049

[jpy70209-bib-0061] Phillips, S. J. , Anderson, R. P. , & Schapire, R. E. (2006). Maximum entropy modeling of species geographic distributions. Ecological Modelling, 190(3–4), 231–259. 10.1016/j.ecolmodel.2005.03.026

[jpy70209-bib-0062] Phillips, S. J. , & Dudík, M. (2008). Modeling of species distributions with Maxent: New extensions and a comprehensive evaluation. Ecography, 31(2), 161–175. 10.1111/j.0906-7590.2008.5203.x

[jpy70209-bib-0063] R Core Team . (2024). R: A language and environment for statistical computing. R Foundation for Statistical Computing. Https://www.R‐project.org/

[jpy70209-bib-0064] Rendina, F. , Falace, A. , Alongi, G. , Buia, M. C. , Neiva, J. , Appolloni, L. , Marletta, G. , & Russo, G. F. (2023). The lush Fucales underwater forests off the Cilento coast: An overlooked Mediterranean biodiversity hotspot. Plants, 12(7), 1497. 10.3390/plants12071497 PMC10096796 37050123

[jpy70209-bib-0065] Riera, R. , Sangil, C. , & Sansón, M. (2015). Long‐term herbarium data reveal the decline of a temperate‐water algae at its southern range. Estuarine, Coastal and Shelf Science, 165, 159–165. 10.1016/j.ecss.2015.05.008

[jpy70209-bib-0066] Roberts, D. R. , Bahn, V. , Ciuti, S. , Boyce, M. S. , Elith, J. , Guillera‐Arroita, G. , Hauenstein, S. , Lahoz‐Monfort, J. J. , Schröder, B. , Thuiller, W. , Warton, D. I. , Wintle, B. A. , Hartig, F. , & Dormann, C. F. (2017). Cross‐validation strategies for data with temporal, spatial, hierarchical, or phylogenetic structure. Ecography, 40(8), 913–929. 10.1111/ecog.02881

[jpy70209-bib-0067] Román, S. , & Vázquez, R. (2025). Assessment of the *Rugulopteryx okamurae* invasion in northeastern Atlantic and Mediterranean bioregions: Colonisation status, propagation hypotheses and temperature tolerance thresholds. Marine Environmental Research, 207, 107093. 10.1016/j.marenvres.2025.107093 40120426

[jpy70209-bib-0068] Rosas‐Guerrero, J. , Carmona, R. , De La Rosa, J. , Zanolla, M. , & Altamirano, M. (2025). A population dynamics approach to understand the invasiveness of the seaweed *Rugulopteryx okamurae* (Ochrophyta, Dictyotales). NeoBiota, 97, 1–17. 10.3897/neobiota.97.137873

[jpy70209-bib-0069] Rueda, J. L. , Mena‐Torres, A. , Gallardo‐Núñez, M. , González‐García, E. , Martín‐Arjona, A. , Valenzuela, J. , García‐Ruiz, C. , González‐Aguilar, M. , Mateo‐Ramírez, Á. , García, M. , Sayago‐Gil, M. , & Vázquez, J. T. (2023). Spatial distribution and potential impact of drifted thalli of the invasive alga *Rugulopteryx okamurae* in circalittoral and bathyal habitats of the Northern Strait of Gibraltar and the Alboran Sea. Diversity, 15(12), 1206. 10.3390/d15121206

[jpy70209-bib-0070] Ruete, A. (2015). Displaying bias in sampling effort of data accessed from biodiversity databases using ignorance maps. Biodiversity Data Journal, 3, e5361. 10.3897/BDJ.3.e5361 PMC4549634 26312050

[jpy70209-bib-0071] Sales, M. , & Ballesteros, E. (2010). Long‐term comparison of algal assemblages dominated by *Cystoseira crinita* (Fucales, Heterokontophyta) from Cap Corse (Corsica, North Western Mediterranean). European Journal of Phycology, 45(4), 404–412. 10.1080/09670262.2010.498585

[jpy70209-bib-0072] Sales, M. , Cebrian, E. , Tomas, F. , & Ballesteros, E. (2011). Pollution impacts and recovery potential in three species of the genus *Cystoseira* (Fucales, Heterokontophyta). Estuarine, Coastal and Shelf Science, 92(3), 347–357. 10.1016/j.ecss.2011.01.008

[jpy70209-bib-0073] Simon‐Nutbrown, C. , Hollingsworth, P. M. , Fernandes, T. F. , Kamphausen, L. , Baxter, J. M. , & Burdett, H. L. (2020). Species distribution modeling predicts significant declines in coralline algae populations under projected climate change with implications for conservation policy. Frontiers in Marine Science, 7, 575825. 10.3389/fmars.2020.575825

[jpy70209-bib-0074] Soberon, J. , & Peterson, A. T. (2005). Interpretation of models of fundamental ecological niches and species' distributional areas. Biodiversity Informatics, 2, 1–10. 10.17161/bi.v2i0.4

[jpy70209-bib-0075] Srivastava, V. , Lafond, V. , & Griess, V. C. (2019). Species distribution models (SDM): Applications, benefits and challenges in invasive species management. CABI Reviews, 14, 1–13. 10.1079/PAVSNNR201914020

[jpy70209-bib-0076] Sudo, K. , Watanabe, K. , Yotsukura, N. , & Nakaoka, M. (2020). Predictions of kelp distribution shifts along the northern coast of Japan. Ecological Research, 35(1), 47–60. 10.1111/1440-1703.12053

[jpy70209-bib-0077] Sverdrup, H. U. (1953). On conditions for the vernal blooming of phytoplankton. ICES Journal of Marine Science, 18(3), 287–295. 10.1093/icesjms/18.3.287

[jpy70209-bib-0078] Thibaut, T. , Pinedo, S. , Torras, X. , & Ballesteros, E. (2005). Long‐term decline of the populations of Fucales (*Cystoseira* spp. and *Sargassum* spp.) in the Albères coast (France, North‐western Mediterranean). Marine Pollution Bulletin, 50(12), 1472–1489. 10.1016/j.marpolbul.2005.06.014 16026805

[jpy70209-bib-0079] Tyberghein, L. , Verbruggen, H. , Pauly, K. , Troupin, C. , Mineur, F. , & De Clerck, O. (2012). Bio‐ORACLE: A global environmental dataset for marine species distribution modelling. Global Ecology and Biogeography, 21(2), 272–281. 10.1111/j.1466-8238.2011.00656.x

[jpy70209-bib-0080] Valiela, I. , McClelland, J. , Hauxwell, J. , Behr, P. J. , Hersh, D. , & Foreman, K. (1997). Macroalgal blooms in shallow estuaries: Controls and ecophysiological and ecosystem consequences. Limnology and Oceanography, 42(5part2), 1105–1118. 10.4319/lo.1997.42.5_part_2.1105

[jpy70209-bib-0081] Warren, D. L. , & Seifert, S. N. (2011). Ecological niche modeling in Maxent: The importance of model complexity and the performance of model selection criteria. Ecological Applications, 21(2), 335–342. 10.1890/10-1171.1 21563566

[jpy70209-bib-0082] Wernberg, T. , Bennett, S. , Babcock, R. C. , De Bettignies, T. , Cure, K. , Depczynski, M. , Dufois, F. , Fromont, J. , Fulton, C. J. , Hovey, R. K. , Harvey, E. S. , Holmes, T. H. , Kendrick, G. A. , Radford, B. , Santana‐Garcon, J. , Saunders, B. J. , Smale, D. A. , Thomsen, M. S. , Tuckett, C. A. , & Wilson, S. (2016). Climate‐driven regime shift of a temperate marine ecosystem. Science, 353(6295), 169–172. 10.1126/science.aad8745 27387951

