# Supplementary material for: Modeling of the geographical distribution of two forest‐forming fucoids in the Strait–Alboran demarcation (southern Iberian Peninsula): Implications for conservation and restoration
Source: J Phycol. 2026 Jul 31;62(5):1606–17. doi: 10.1111/jpy.70209 (PMC13659351; doi:10.1111/jpy.70209)
Supplement: Supplementary file 1 [file JPY-62-1606-s001.pdf]

Supplementary information for:

Modeling of the geographical distribution of two forest-forming fucoids in the Strait-Alboran demarcation (Southern Iberian Peninsula): implications for conservation and restoration

Pablo Pérez-Castro[1], Elena Bañares-España[1]*, Antonio Flores-Moya[1]*# and Ignacio J. Melero-Jiménez[1]*#

*Corresponding authors: elbaes@uma.es, floresa@uma.es, imelero@uma.es

# These authors contributed equally

[1] Departamento de Botánica y Fisiología Vegetal, Facultad de Ciencias, Universidad de Málaga, Campus de Teatinos s/n, Málaga 29071, Spain

Environmental Predictor collinearity

Prior to model calibration, collinearity among candidate environmental predictors was assessed to avoid parameter instability and misleading ecological inference in the species distribution models. Pairwise Pearson correlation coefficients were calculated among all candidate predictors using 10,000 randomly sampled grid cells within the global calibration domain. Predictors showing strong collinearity ($|r| > 0.7$) were not retained simultaneously, following established best-practice recommendations for species distribution modeling (Dormann et al., 2013; Elith et al., 2011).

To ensure methodological consistency and transferability between present and future projections, two additional constraints were applied. First, only long-term climatological mean predictors were considered for model calibration, avoiding the combination of different temporal statistics (e.g., mean together with minimum or maximum layers of the same variable). Second, only predictors available for both present-day and future SSP environmental layers were retained to ensure comparability between calibration and projection environments (Elith & Leathwick, 2009).

When strong collinearity was detected ($|r| > 0.7$), the predictor with greater ecological interpretability for shallow coastal macroalgae and stronger conceptual relevance to nearshore physical processes was retained. Highly correlated nutrient variables (e.g., nitrate and phosphate) were therefore not included simultaneously. Candidate predictors were evaluated according to their ecological relevance across several functional categories, including physical drivers (e.g., temperature, mixed layer depth, circulation), chemical conditions (e.g., pH), nutrient availability, and ecosystem productivity proxies.

After correlation filtering, the final predictor set (identical for both species) included the following variables:

- thetao_mean – sea surface temperature
- mlotst_mean – mixed layer depth
- sws_mean – sea water velocity
- swd_mean – sea water direction
- ph_mean – seawater pH
- chl_mean – chlorophyll-a concentration
- clt_mean – cloud cover
- si_mean – silicate concentration

Excluded variables included tas_mean, o2_mean, phyc_mean, po4_mean, no3_mean, and so_mean, which were removed due to strong collinearity or redundancy with retained predictors**.** Although dfe_mean was retained after the initial collinearity screening, it was excluded from the final species-specific analyses because it showed negligible environmental variance within the accessible calibration domain.

The full pairwise correlation matrix of candidate predictors is provided in Figure S1**,** where retained variables are distinguished from excluded predictors. Detailed information on all candidate and retained predictors is reported in Supplementary Table S3**.**

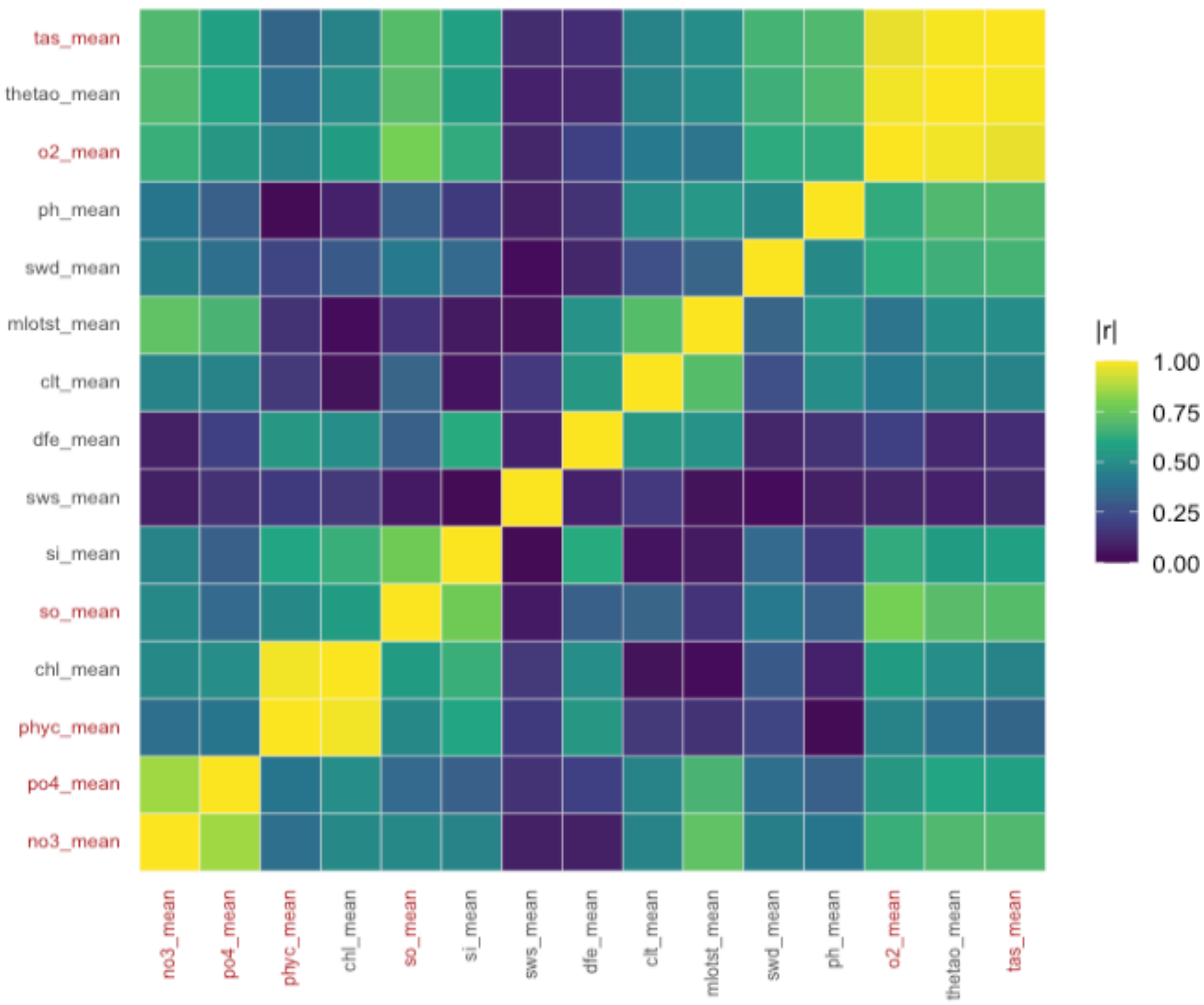

Figure S1. Pairwise Pearson correlation matrix (|r|) among candidate environmental predictors sampled within the species-specific calibration area (M). Variables retained after correlation filtering are shown in black; excluded predictors (|r| > 0.7 or not consistently available across future scenarios) are shown in red. Abbreviations are: thetao_mean = sea surface temperature (°C); mlotst_mean = mixed layer depth (m); sws_mean = sea water velocity (m s^{-1}); swd_mean = sea water direction (degrees); ph_mean = seawater pH; dfe_mean = dissolved iron concentration (µmol m^{-3}); chl_mean = chlorophyll-a concentration (mg m^{-3}); clt_mean = total cloud cover (fraction); no3_mean = nitrate concentration (µmol m^{-3}); po4_mean = phosphate concentration (µmol m^{-3}); si_mean = silicate concentration (µmol m^{-3}); o2_mean = dissolved oxygen concentration (mmol m^{-3}); phyc_mean = primary productivity (mg C m^{-3} day^{-1}); so_mean = salinity (psu); tas_mean = air temperature (°C).

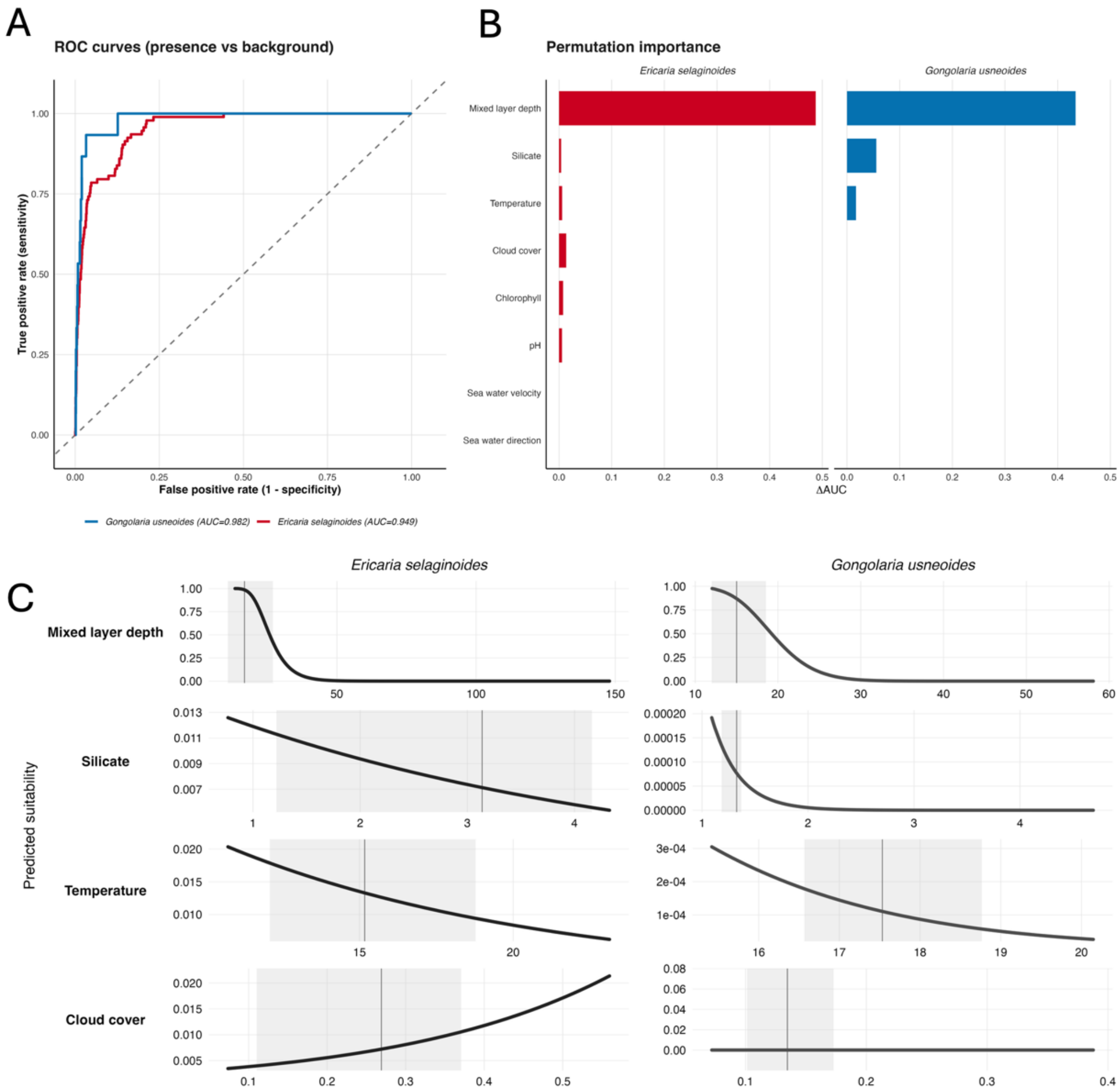

Figure S2. Model performance and variable importance for *Ericaria selaginoides* and *Gongolaria usneoides*. The left panel shows receiver operating characteristic (ROC) curves comparing occurrence records against background points for the final MaxEnt models. Both species exhibited high discriminatory performance under cross-validation, with mean validation AUC values of 0.949 for *E. selaginoides* (spatial block) and 0.982 for *G. usneoides* (leave-one-out jackknife). The dashed diagonal line represents the performance expected under random prediction. The right panel shows permutation importance of environmental predictors, expressed as the decrease in model performance (ΔAUC) after random permutation of each variable. For both species, mixed layer depth is the most influential predictor, followed by cloud cover and sea surface temperature in *E. selaginoides*, and silicate concentration and sea-water temperature in *G. usneoides*. All remaining predictors contributed only marginally to model performance relative to mixed layer depth. The C panel shows marginal response curves for the four most influential predictors in each species. Curves represent model-predicted habitat suitability (cloglog output) across the observed range of each predictor, while holding all other variables at their median values. Grey bands indicate the central occupied range of presences (10th–90th percentile), and vertical lines represent median values. Consistent with permutation importance results, suitability declines sharply with increasing mixed layer depth in both species, whereas responses to other variables are comparatively weak or gradual, indicating that environmental constraints are strongly dominated by vertical water-column structure rather than by secondary physicochemical factors.

Environmental gradient occupancy analysis

To better interpret predictors showing high permutation importance despite relatively shallow marginal response curves, we quantified the proportion of the available environmental gradient occupied by each species within the species-specific accessible area (M).

For each species, the accessible area (M) was defined as the geographic bounding box of occurrence records expanded by a 2° buffer in all directions, matching the calibration domain used for model fitting. For each retained environmental predictor, the total environmental range available within M was calculated from all raster cells in the cropped environmental layer. The environmental range occupied by the species was then calculated from predictor values extracted at presence locations.

Environmental occupancy was expressed as:

$$\text{Occupancy (\%)} = \frac{\max(\text{presence values}) - \min(\text{presence values})}{\max(\text{raster values in } M) - \min(\text{raster values in } M)} \times 100$$

Lower percentages indicate that occurrence records occupy a narrow fraction of the environmental gradient available within M, whereas higher percentages indicate broader environmental occupancy. Results are shown in Supplementary Figure S3.

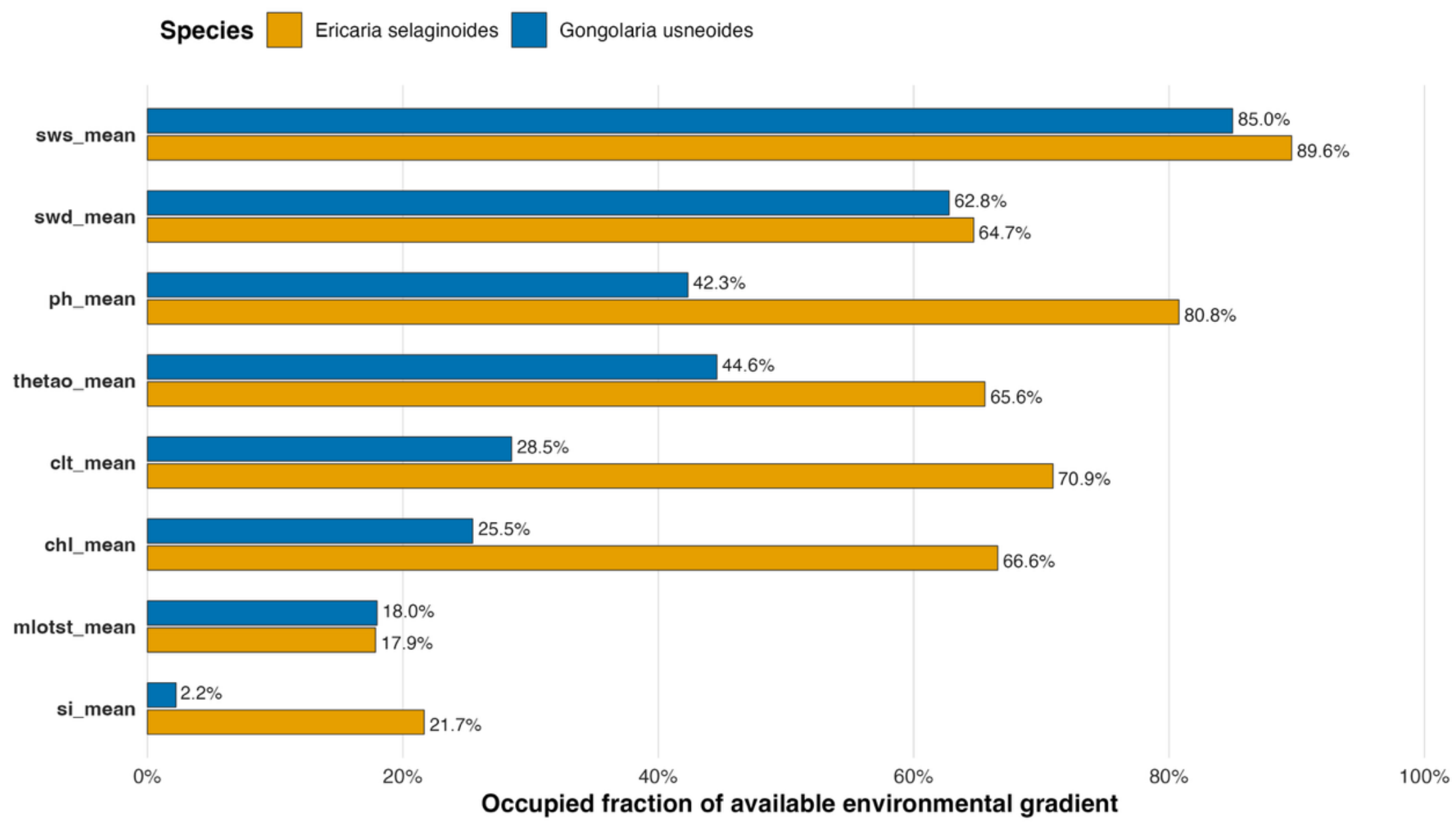

Figure S3. Environmental gradient occupancy within the accessible area (M) for *Ericaria selaginoides* and *Gongolaria usneoides*. Bars represent the proportion of the environmental gradient available within the accessible area (M) that is encompassed by the observed occurrence range for each retained environmental predictor. Values indicate the percentage of the environmental space occupied by each species along the corresponding environmental gradient. Higher percentages indicate broader environmental occupancy relative to the available conditions within M, whereas lower values indicate more restricted environmental ranges. Overall, *E. selaginoides* occupies a broader fraction of the available environmental gradients, whereas *G. usneoides* shows more restricted environmental occupancy, particularly for mixed layer depth.

Supplementary Tables S1–S3 are provided as .csv files.

Table S1. GBIF datasets contributing occurrence records for *Ericaria selaginoides* and *Gongolaria usneoides* used in model calibration after spatial thinning.

Table S2. Geographic coordinates and metadata of occurrence records used to calibrate the species distribution models after data cleaning and 5-km spatial thinning.

Table S3. Bio-ORACLE environmental datasets retained after correlation filtering and considered as candidate predictors for present and future species distribution modeling.

Table S4. Model calibration settings and optimal parameters selected during ENMeval tuning. The number of presences corresponds to the final dataset used for model calibration after spatial thinning. Models were selected using a hierarchical criterion prioritizing minimum omission rate at the 10th percentile training presence threshold (OR10) and, secondarily, maximum validation AUC.

Species	Presences	Optimal feature class	Regularization multiplier	Validation AUC (mean ± SD)	OR10 (mean ± SD)
G. usneoides	15	LQ	2.0	0.982 ± 0.031	0.133 ± 0.352
E. selaginoides	93	L	4.0	0.949 ± 0.020	0.122 ± 0.116
